# A novel germline *CDH23* variant as a likely cause of an ultra-giant prolactinoma

**DOI:** 10.1186/s13023-025-04161-w

**Published:** 2026-01-29

**Authors:** Eman Albasri, Balgees Alghamdi, Avaniyapuram Kannan Murugan, Eyas Othman, Sarah Alotaibi, Mohammad Anas Dababo, Ahmed Alfares, Ali S. Alzahrani

**Affiliations:** 1 Department of Medicine, King Faisal Specialist Hospital and Research Centre, P. O. Box 3354, Riyadh, 11211 Saudi Arabia; 2 Department of Molecular Oncology, King Faisal Specialist Hospital and Research Centre, P. O. Box 3354, Riyadh, 11211 Saudi Arabia; 3 Department of Otolaryngology/Head and Neck Surgery, King Faisal Specialist Hospital and Research Centre, P. O. Box 3354, Riyadh, 11211 Saudi Arabia; 4 Department of Pathology and Laboratory Medicine, King Faisal Specialist Hospital and Research Centre, P. O. Box 3354, Riyadh, 11211 Saudi Arabia; 5 Genome Medicine Center of Excellence, King Faisal Specialist Hospital and Research Centre, P. O. Box 3354, Riyadh, Saudi Arabia 11211; 6 College of Medicine, Alfaisal University, P. O. Box 3354, Riyadh, 11533 Kingdom of Saudi Arabia

**Keywords:** Prolactinoma, Pituitary adenoma, Giant prolactinoma, CDH23, Prolactin

## Abstract

Giant prolactinomas are defined as pituitary adenomas (PAs) ≥ 4 cm with plasma prolactin level > 1000 ng/ml with no other co-secretory component. The reasons for development of giant prolactinomas are not clear but genetics play an important pathogenic mechanism in some PAs. In this report, we describe a middle-aged woman who incidentally was found to have an infiltrative giant prolactinoma involving the sellar, supra- and parasellar regions and occupying most of the middle fossae of the skull extending all the way to the occipital and upper cervical regions. Anteriorly, it extends to the sphenoid and parasellar sinuses, nasopharynx and nasal cavities. It was initially thought to be a nasopharyngeal cancer but biopsy from a protruding component from the right nostril showed that it was a prolactinoma. Prolactin level after several dilutions was extremely high at 277,500 ng/ml (normal range 3.4–24.1 ng/ml). Surprisingly, her pituitary function evaluation showed only mild central hypothyroidism [(FT4 11.4 pmol/l (normal range 12–22) and TSH 1.9 mU/l (normal range 0.27–4.2)] and hypogonadotropic hypogonadism (E2 47 pmol/l, LH 1.9 u/l, FSH 5.9 u/l). Cosyntropin stimulation test was normal suggesting normal pituitary adrenal axis but insulin-induced hypoglycaemia test was not performed. In retrospect, the patient reported chronic nasal congestion and snoring, headaches on/off and deterioration in her hearing and visual acuity over the last few years. She ascribed these symptoms to sinusitis and advancing age, respectively. Whole exome sequencing revealed a novel variant in *CDH23* (NM_022124.6:c.2621C > A, p.Ala874Asp), a gene that has been previously reported to be associated with PAs. The patient was treated with small doses of cabergoline (0.5 mg twice weekly) and reported remarkable improvement in her symptoms. Radiological evaluation confirmed the significant response of this giant prolactinoma to cabergoline.

## Introduction

Prolactinoma (ORPHA 2965) is a benign prolactin-secreting tumor of the pituitary gland. Giant pituitary neuroendocrine tumors, defined as pituitary adenomas (PAs) ≥ 4 cm in size with plasma prolactin level > 1000 ng/ml and absence of co-secretion of other hormones, are rare occurring in 1–5% of all cases of prolactinomas [1–4]. They are more common in males and have variable presentations [3, 5]. Giant PAs may invade the cavernous sinuses, brain and skull base and can be complicated by CSF leak, cranial nerve palsy, visual field defects, neurological deficits and craniospinal instability [5–9]. Systematically, giant prolactinomas might be associated with the usual symptoms of hyperprolactinemia such as decreased libido and gynecomastia in men, amenorrhea and galactorrhea in women and symptoms of partial or panhypopituitarism due to damage of the adjacent normal pituitary tissue by the expanding PA [5].

It is not clear why some prolactinomas become giant while the majority are either microadenomas (≤ 1 cm) or macroadenomas (>1 cm but less in size than giant) [2–4]. The possibility of variable genetic drivers could be a reason for this disparity in size of prolactinoma [10–13]. Time and rate of growth are other factors that may explain the different sizes of prolactinomas at the time of detection.

In recent years, genetics have been shown to play an important role in the pathogenesis of some pituitary tumors [12, 14]. Although PAs were considered sporadic tumors for a long time, studies over the last decade have reported that a significant proportion of PAs carry germline (about 5%), mosaic (<1%) or somatic genetic alterations (~ 40%) (reviewed in reference [15]). Germline variants in *CDH23* have been reported to be associated with PAs [11, 12]. However, data on the role of this gene in PAs are limited. In this report, we describe a middle-aged lady, incidentally found to have a huge giant prolactinoma involving the pituitary gland, cavernous sinuses, suprasellar and parasellar space, middle and posterior cranial fossae, sphenoid and paranasal sinuses, nasopharynx and nostrils. She was found to have a novel pathogenic *CDH23* variant that is likely the underlying genetic cause of this giant prolactinoma.

## Case presentation

**Fig. 1:**
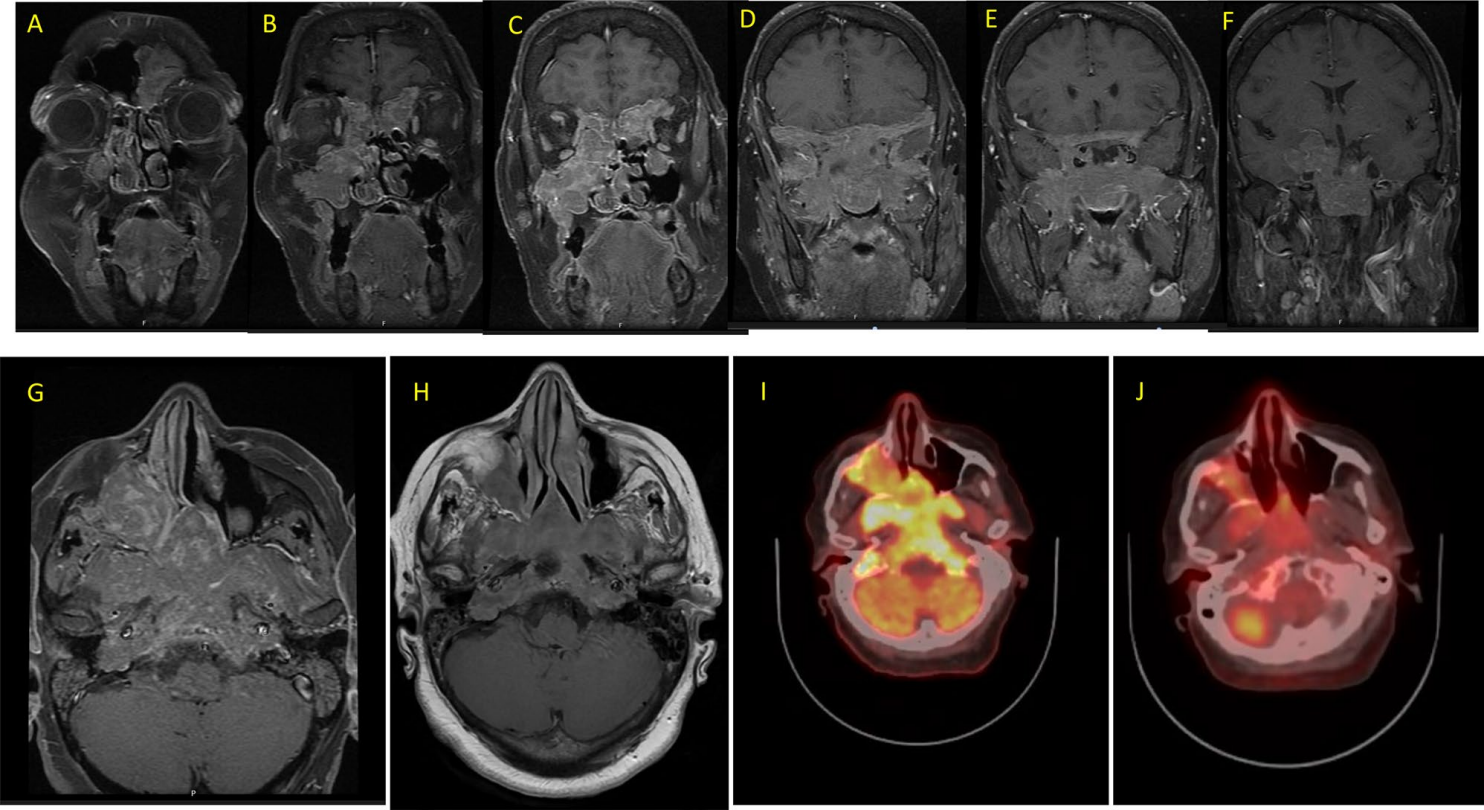
The upper panel (**A**–**F**) are serial coronal sections of a gadolinium-enhanced T1 images showing a huge enhancing mass involving the sellar, parasellar and suprasellar region and extending inferiorly to the sphenoid and paranasal sinuses, nasopharynx and nasal cavity, especially on the right side. It extends laterally and posteriorly to the middle and posterior fossa. In the lower panel, these axial sections show the mass before starting therapy (**G**) and after 7 months on cabergoline therapy (**H**) showing a major reduction in the size and enhancement. The same is also demonstrated on FDG PET CT scans done before (I) and 7 months after starting cabergoline therapy (**J**) revealing a major reduction in the size and avidity of the giant prolactinoma that this patient had

A 53-year-old woman with a background history of type 2 diabetes mellitus, hypertension, and stage IV chronic renal insufficiency, accidentally fell down and sustained trauma to the back of her head. This was followed by a single episode of vomiting. Imaging studies, including CT and MRI of the brain, revealed a huge mass extensively involving the sella, suprasellar and parasellar space, cavernous sinuses, and middle fossae. The mass measured 6.5 cm craniocaudal, 8 cm transeverse, and 7 cm anteroposterior and extended inferiorly to the sphenoid and parasellar sinuses, and nasopharynx and was visible at the right nostril (Fig. 1A-G). An ^18^F- Fluorodeoxyglucose positron emission tomography merged with computed tomography (FDG PET CT) scan showed a heterogeneous but highly avid uptake in the mass (Fig. 1-I).

On direct questioning, the patient reported chronic headaches, snoring, right-sided nasal obstruction, hyposmia, impaired memory and bilateral hearing loss, which had progressively worsened over the past four years. She had been using a hearing aid since 2021. She also noticed decreased visual acuity over the last few years. She thought that these symptoms were due to chronic sinusitis and advancing age. She denied history of nausea, vomiting, double vision, weakness, sensory loss, or seizure. No history of nasal discharge, facial pressure, otorrhea, dysphagia, choking, cough or any constitutional symptoms such as fever, night sweats, or weight loss. There was no previous history of malignancy or exposure to irradiation. She reported no history of galactorrhea. Her menarche started at the age of 13 years with regular menses until her late twenties, when she started to have oligomenorrhea, and was diagnosed with hyperprolactinemia at that time for which she was prescribed medical treatment for a few months but no further investigations. Her menstrual periods had ceased more than 15 years ago at age 38 years. The patient has never been married and has no children. Family history was negative for pituitary or other tumors, and no history of hearing impairment or any known syndrome in the family.

**Fig. 2:**
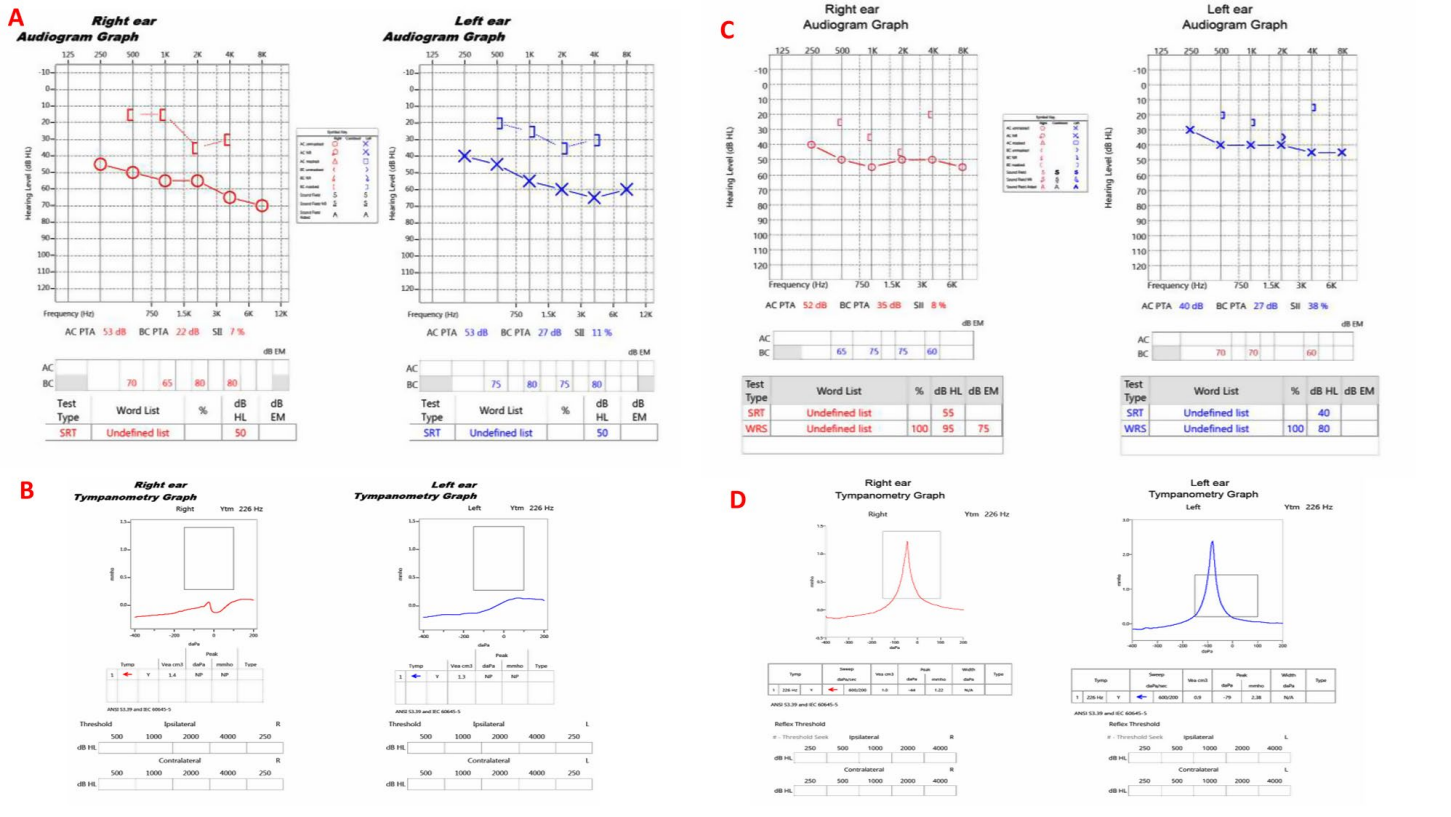
Pure-tone audiograms before and 2.5 years after treatment of a large pituitary adenoma. Panels **A** and **B** show the pre-treatment audiograms demonstrating sensorineural hearing loss, more pronounced at higher frequencies. Panels **C** and **D** represent the post-treatment (Cabergoline) audiograms, showing marked improvement in hearing thresholds and speech discrimination, particularly in the mid- and high-frequency ranges

Physical examination revealed that she was stable, body mass index 27.8 kg/m2, pulse rate 84 per minute, and blood pressure 146/89 mm Hg with no orthostatic hypotension. No hirsutism, acne, or expressible galactorrhea. No visual field defects, diplopia, sensory or motor deficits. No gross visual field defects on bedside confrontation test. She did not have any features of Cushing syndrome or acromegaly. Her systemic examination was otherwise normal. Audiological assessment revealed bilateral moderate to severe mixed (sensorineural and conduction) hearing loss (Fig. 2a and b). Baseline ophthalmologic evaluation performed prior to therapeutic intervention and demonstrated normal appearance of the optic nerve and retina without evidence of diabetic or hypertensive retinopathy and no signs of retinitis pigmentosa or optic atrophy. Perimetry examination showed mild nonspecific decrease in light sensitivity. Formal evaluation of visual field was scheduled but the patient failed to attend her appointment.

Initially, the mass was suspected to be a nasopharyngeal cancer, however a bedside biopsy of a protruding component of the tumor from the right nostril unexpectedly revealed pituitary tissue that was strongly positive for prolactin. The undiluted serum prolactin level was 47 ng/ml (normal 3.4–24), and after several dilutions for possible hook effect, the level rose to 277,500 ng/ml. Baseline fasting GH was normal at 4.7 ng/ml (normal < 9.9 ng/ml) and plasma insulin-like growth factor-1 (IFG-1) level was slightly elevated but oral glucose tolerance test was not done. Other anterior pituitary hormones including random GH, morning cortisol, ACTH, and TSH were within normal limits. However, FT4 was mildly low consistent with mild central hypothyroidism (Table 1). A short cosyntropin test showed an adequate adrenal response to synthetic ACTH (Table 1). However, central adrenal insufficiency cannot be definitely excluded by this test. An insulin hypoglycemia test was not done as it was felt to be non-essential and might carry a risk of inducing seizure or other complications of hypoglycemia.

**Table 1:** Summary of the hormonal evaluation at the time of diagnosis before starting cabergoline treatment and 1 year later

Hormone/Test	Normal Range	Before Treatment	1 year on cabergoline
Prolactin	3.4–24.1 ng/ml	277500	1.13
IGF-1	93–245 ng/ml	253	88.9
FSH	3.5–12.5 IU/L	5.9	59
LH	2.4–12.6 IU/L	1.9	72
E2	Up to 201 pmol/L	47	< 18
TSH	0.27–4.2 mU/l	1.9	1.7
FT4	12–22 Pmol/L	11.4	15.8
ACTH	5–60 ng/L	16.2	39
Cortisol AM	140–690 nmol/L	213	402
Cortisol 30 min. after ACTH injection		501	-
Cortisol 60 min after ACTH injection		449	-

**Fig. 3:**
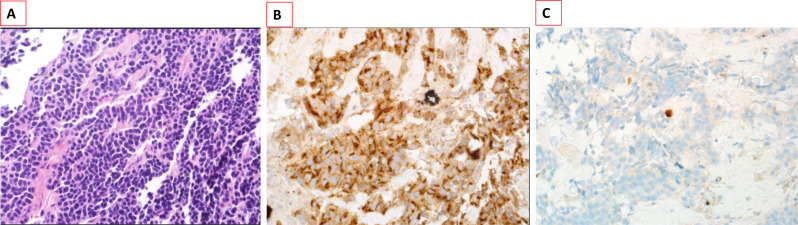
Histopathological examination of a biopsy from the right nasal cavity component of the tumor showing: **A**. sheets of monomorphic small round basophilic cells with diffuse pattern (H&E stain, 400X), **B**. Immunohistochemistry for prolactin showing strong positivity (prolactin stain, 400X) and **C**. Ki67 stain showing a low proliferation index of < 1% (400X)

Pathological examination was repeated from a deeper biopsy of the right nasal septum (Vomer) and confirmed the diagnosis of a lactotroph PA (Fig. 3). Immunohistochemical staining showed strong positivity for CAM5.2, synptophysin, prolactin, and CD56, with low Ki-67 of 1% (Fig. 3). The tumor was negative for CK5/6, CK7, TTF1, CD45, TSH, GH, FSH, LH and ACTH.

The patient was treated with low dose cabergoline 0.25 mg twice weekly to avoid the risk of rapid tumor shrinkage and apoplexy or CSF leak, along with levothyroxine 50 mcg daily. Two months later, cabergoline dose was increased to 0.5 mg twice weekly resulting in noticeable improvement in hearing, vision and nasal congestion.

**Fig. 4:**
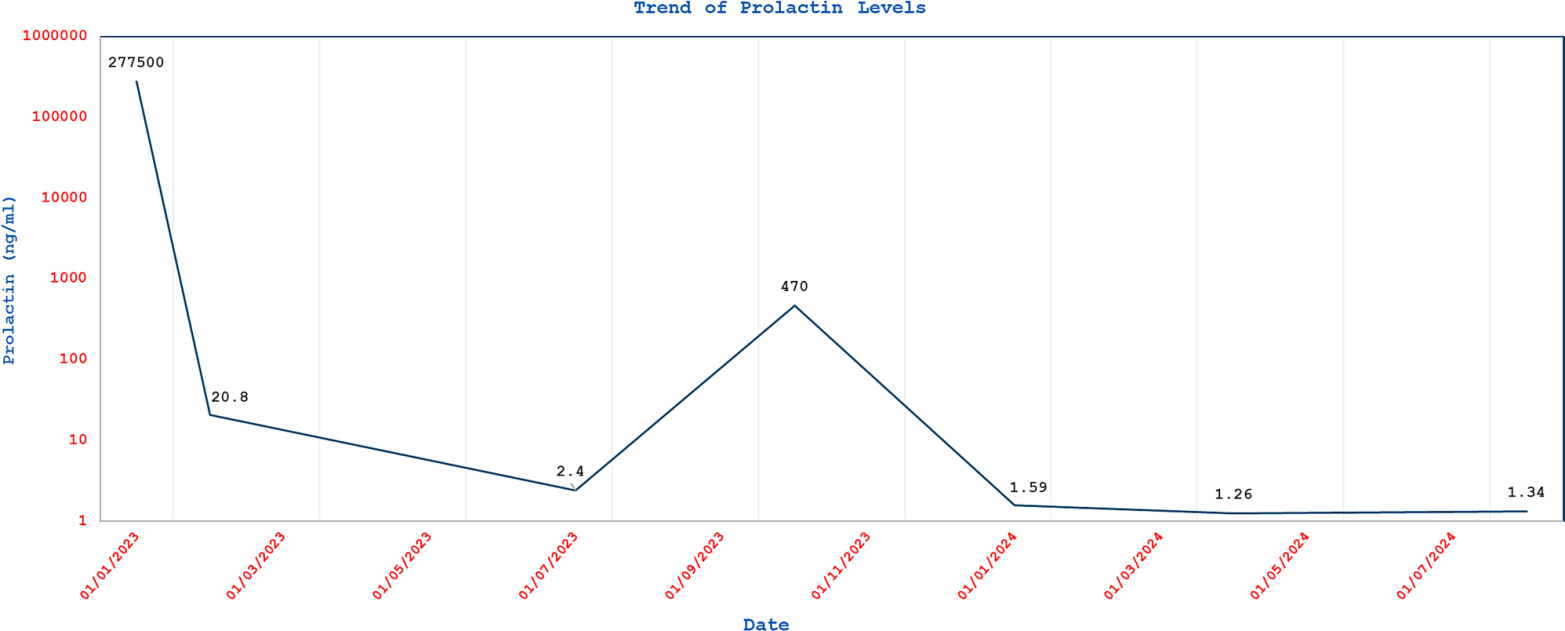
A chart showing prolactin levels over time. The prolactin level briskly dropped from very high level of 277,500 ng/ml before starting cabergoline to 20 ng/dl after only about 1 month of therapy. The level remained suppressed until the patient stopped cabergloine for 3 months in late July 2023 when it rose to 470 ng/dl. However, it did not increase to the same original level, probably reflecting the induced apoptosis and suppression of the tumor by the 7 month-cabergoline therapy. Resumption of cabergoline therapy normalized prolactin level again

One month later, prolactin level dropped to 20 ng/ml (unchanged with several dilutions) and remained low at 2.5 ng/ml 6 months later which was confirmed with a dilution factor of 100 (Fig. 4).

Central adrenal insufficiency was essentially ruled out with an adequate response to a short cosyntropin test. Follow up MRI and FDG PET CT scan 7 months post treatment showed significant reduction in the size and activity of the diffuse infiltrative lesion (Fig. 1H and J). After holding cabergoline for three months by the patient, prolactin level increased to 470 ng/ml (Fig. 4). Therefore, cabergoline was resumed at a dose of 0.5 mg three times weekly. Three months later, prolactin level was notably low at 1.59 and 1.3 ng/ml (Fig. 4). Consequently, the cabergoline dose was reduced back to 0.5 mg twice weekly. During a recent follow up visit to the clinic, the patient reported marked improvement in her headaches, sense of smell, memory, concentration, and hearing. Furthermore, she is no longer using her hearing aids. Repeated audiogram in 2025 (on cabergoline for about 2.5 years) showed marked improvement in hearing thresholds and speech discrimination (Fig. 2c and d). Interestingly, she also reported spontaneous resumption of her period for few months following treatment with cabergoline. However, her period eventually stopped, and hormonal profile confirmed that she had entered natural menopause with high FSH, LH, low estradiol and normal prolactin (Table 1).

### Molecular testing

**Fig. 5:**
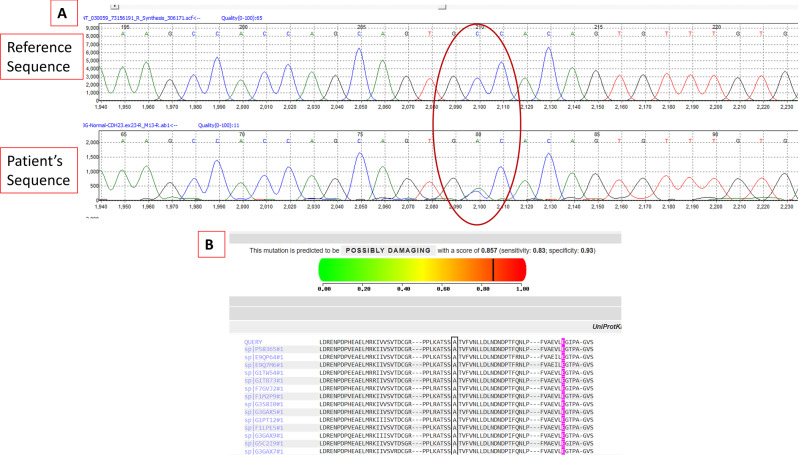
A chromatogram (**A**) of a segment of *CDH23* exon 17 showing a heterozygous variant (NM_022124.6:c.2621C > A) changing the amino acid at this codon from Alanine to Aspartic acid (p.Ala874Asp). This variant is predicted to be pathogenic by most *in silico* analysis tools. The analysis by PolyPhen2 is shown along with the conservation among different species of the alanine residue at this position (**B**)

The study was approved by the Institutional Review Board of the King Faisal Specialist Hospital & Research Centre, Riyadh, Saudi Arabia (RAC# 2130012). An informed consent was obtained from the patient. Whole exome ssequencing (WES) was performed on genomic DNA isolated from the peripheral leucocytes. The DNA isolation, WES and bioinformatics analysis were performed as previously described [12]. This identified a novel heterozygous variant in *CDH23* (NM_022124.6:c.2621C > A, p.Ala874Asp). The identified variant was confirmed by Sanger sequencing (Fig. 5a). It was designated variant of unknown significance (VUS) according to the American College of Medical Genetics and Genomics (ACMG) [16]. This variant has not been reported in gnomAD exome and genome databases, ClinVar or Human Gene Mutation Database (HGMD). It is predicted to be moderately pathogenic by FATHMM-XF, AlphaMissence, EIGEN, EIGEN PC, FATHMM-MKL, MutPred, and SIFT4G. It is possibly damaging by PolyPhen2 with a score of 0.857 (Fig. 5b). The alanine residue at this position is highly conserved between different species (Fig. 5b). Other known PA-associated genes did not reveal any potential pathogenic, likely pathogenic or of unknown significance variants. Genes that were particularly analyzed included *AIP, GNAS, MEN1, SDHx, DICER1, PRKAR1A, NF1, USP8, USP48, CDKN1B, ATRX, DAXX, PIK3CA, PTEN, BRAF, TERT, TP53, SF3B1 and KMT2D.*

## Discussion

In this report, we describe an unusually huge giant prolactinoma discovered incidentally on a CT scan of the head done for evaluation of an accidental fall. In retrospect, the patient was having symptoms that she did not consider significant. These symptoms included on/off headaches, hearing and visual impairment that she thought to be related to age and nasal congestion and snoring that she ascribed to possible sinusitis. Her evaluation demonstrated the expected hook effect of giant prolactinoma with the initial prolactin level of only 47 ng/ml (normal range 3.4–24.1) increasing with several dilutions to 277,500 ng/ml. Surprisingly, her pituitary function was not significantly affected with only inappropriately normal gonadotropins for her postmenopausal status and mild central hypothyroidism. IGF-1 was slightly elevated initially, which may suggest a minor component of somatolactotroph adenoma but decreased to below normal range without specific therapy except small doses of cabergoline. The hypothalamic adrenocortical axis was probably normal with normal baseline and post-cosyntropin cortisol levels although it was not tested by insulin-induced hypoglycemia test. In addition, she did not have visual field defect on perimetry. This giant prolactinoma showed an excellent response to cabergoline with remarkable improvement in the patient’s symptoms, brisk normalization of prolactin, reduction in size and enhancement of PA on MRI and significant reduction of FDG avidity on FDG PET CT scan.

Giant prolactinomas are rare accounting for 1–5% of all prolactinomas [2–6]. In a systematic review and detailed analysis of giant prolactinomas, Billion L. et al. included 196 adult patients reported in the literature with prolactinomas ≥ 40 mm, prolactin level > 1000 ng/ml and no concomitant secretion of growth hormone or ACTH [5]. The median age was 38 years and the female to male ratio was 1:3.6. The median tumor diameter was 53 mm with a range of 43 to 69 mm. Pituitary deficiency was present in 91% of cases and central hypogonadism was the most common. The treatment included dopamine agonists in 82% of cases, leading to normoprolactinemia in 51%, tumor shrinkage in 88% and visual improvement in 85% of cases. Surgery was performed in 29% of cases but all patients continued to have persistent hyperprolactinemia and tumor remnant [5]. Shahbazi et al. described a 39-year-old man who developed quadriparesis and paresthesia and was found to have a huge prolactinoma (99×72× 57 mm) extending from the pituitary gland to the occipital region causing cranial displacement and central herniation with myelopathy. The patient needed urgent occipital craniotomy followed later by occipitocervical fixation and cabergoline treatment [7]. The authors summarized 5 similar cases reported in the literature since 1979 [7]. All of them needed occipitocervical fixation and fusion [7]. While all of these cases had posterior extension towards the occipital region, our patient’s giant PA had also posterior extension to the occipital area but the extension was mostly anterior in location occupying the sella and suprasellar region and the middle fossae and inferiorly to the sphenoid and paranasal sinuses, nasopharynx and nasal cavities. Another report from Saudi Arabia described a 64-year-old man who presented with panhypopituitarism, mild facial palsy and right homonymous hemianopia [17]. Prolactin level was 7896 ng/ml (reference range: 2–20 ng/ml). MRI of the brain showed 5.8 × 6 × 5.5 cm giant PA. He initially refused surgery and responded to dopamine agonists but eventually presented with obstructive hydrocephalous and needed insertion of ventricular catheter and craniotomy for tumor resection. This showed a prolactinoma with Ki67 of 1%-2% [17].

Prolactin level was extremely elevated in our patient at 277,500 ng/ml. This might have been further exacerbated by the stage 4 chronic renal insufficiency this patient had. At this stage of renal impairment, the diminished renal clearance of prolactin together with hypothalamic pituitary dysregulation caused by uremic toxins or altered dopamine inhibition leads to marked elevation in serum prolactin level [18, 19].

Due to the unusually huge size of the prolactinoma in this relatively young patient, we suspected a possible underlying genetic cause. WES showed a novel likely pathogenic variant in *CDH23* (c.2621C > A, p.Ala874Asp). Zhang Q. et al. described a *CDH23* variant (c.4136 G > T, P.Arg1379Leu) co-segregating with PA phenotype in 4 affected members of a family with 4 affected and 17 asymptomatic members [11]. They extended their search for *CHD23* variants to 12 families with familial PAs, 125 patients with sporadic PAs and 260 control subjects. The results showed 33% (4/12 families), 12% (15/125) and 0.8% (2/260) harbored functional variants in *CDH23*, respectively [11]. Unlike our patient, PA with *CDH23* variants reported in that study were smaller and less invasive [11]. Although the PA in our patient was huge, the Ki67 index is quite low (1%) and its response to cabergoline was impressive suggesting that this PA grew slowly over several years but was not necessarily aggressive. This is further suggested by the insidious and subtle symptoms that this patient had and the history of hyperprolactinemia in her late twenties and early menopause at age 38 years. In our recently published study of germline variants in 134 sporadic (non-familial) PAs, 2 patients had *CDH23* novel variants. The first variant (c.906 G > C, p.Asp302Glu) was in a 48-year old man with somatolactotroph pituitary macroadenoma and the second (c.1096 G > A, p.Ala366The) in a 22-year-old-woman with non-functioning macroadenoma [12].

*CDH23* mutations in recessive forms cause non-syndromic autosomal recessive deafness and Usher syndrome, a rare syndrome characterized by sensorineural deafness and retinitis pigmentosa [20, 21]. Our patient had mixed bilateral hearing loss, likely due to the huge PA rather than the *CDH23* variant since it improved with cabergoline therapy and tumor shrinkage. In addition, her retinal examination did not reveal signs of retinitis pigmentosa. The *CDH23* variant she has was heterozygous and not expected to cause full blown autosomal recessive Usher syndrome.

CDH23 is a cadherin superfamily member and is involved in cell-to-cell adhesion [22]. These molecules are glycoproteins that mediate adhesion of cells by a calcium-dependent mechanism [11, 22]. They have been reported to have altered expression in PAs [23–25]. CDH23 also forms a heterodimers with protocadherin-related 15 (PCDH15) whose gene is located near a PA-susceptibility gene locus [26]. These observations suggested that CDH23 is involved in PA tumorigenesis and are further supported by Zhang et al’s study and others [11, 12].

This report has some limitations including the fact that it is a single case report limiting its generalizability. It also lacks the family segregation and functional assessment of the reported *CDH23* variant. Therefore, the pathogenicity of the *CDH23* variant and its contribution to the giant PA found in this patient remains speculative. However, the findings are supported by the previous reports that found *CDH23* variants to be pathogenic in some PAs, the *in silico* analysis highly suggesting the pathogenicity of the current variant and its absence in the public genomic databases including gnomAD, ClinVar and HGMD.

In summary, this report describes a middle-aged woman with a huge giant prolactinoma, likely due to an underlying germline *CDH23* variant. The presentation, size and extension of the PA and response to cabergoline were impressive. The finding of a novel likely pathogenic *CDH23* variant further supports previous reports indicating the involvement of this gene in the pathogenesis of some cases of PA.

## Data Availability

Most of the data related to this case are included in this manuscript. Additional data would be available upon request.
